# Hypertension management in the oldest-old: a survey of physicians in Swedish primary health care

**DOI:** 10.1080/02813432.2025.2549088

**Published:** 2025-08-25

**Authors:** Marjo Berkhout, Kristina Bengtsson Boström, Per Hjerpe, Anna-Lena Östberg

**Affiliations:** a General Practice/Family Medicine, School of Public Health and Community Medicine, Institute of Medicine, Sahlgrenska Academy, University of Gothenburg, Gothenburg, Sweden; b Närhälsan Norrmalm Health Care Centre, Skövde, Sweden; c Centre for Research, Education, Development and Innovation, Primary Health Care, Region Västra Götaland, Skövde, Sweden; d Department of Behavioral and Community Dentistry, Institute of Odontology, Sahlgrenska Academy, University of Gothenburg, Gothenburg, Sweden

**Keywords:** Hypertension treatment, oldest-old, primary health care, survey, general practitioner

## Abstract

**Introduction:**

The prevalence of hypertension (HT) increases with age. Uncertainty remains about how to treat the oldest patients, who often suffer from multimorbidity.

**Aim:**

We explored which factors influence general practitioners’ (GPs’) and GP trainees’ treatment of hypertension among the oldest-old.

**Methods:**

GPs and GP trainees in Sweden were invited to fill out an online survey through announcements in newsletters and closed social media groups.

**Results:**

Of the 577 questionnaires that were initiated, 397 were completed (69%). The respondents stated that acceptable blood pressure ranges were 115–152/61–93 mmHg. Regarding factors influencing choices of HT treatment, all respondents considered patient’s living conditions more important than medical factors, more so by female (80%) than by male physicians (71%, *p* = 0.049), and more by respondents less experienced in primary health care (PHC) (83%) compared to more experienced (74%, *p* = 0.043). Lifestyle recommendations, except dietary advice, were frequently offered (80.4%–91.4%). All respondents identified co-morbidity and cardiovascular risk factors as important for treatment decisions. Respondents with more PHC experience considered HT treatment guidelines more useful than those with less experience (*p* = 0.012). Improved cooperation with other caregivers and a common medication list were prioritised more by female than male respondents.

**Conclusions:**

Both medical factors and living conditions were important for GPs and GP trainees in making HT treatment decisions for the oldest-old. Female and less experienced respondents prioritised living conditions. Organisational changes in HT care for the oldest-old were more important to female respondents.

## Introduction

Hypertension (HT) is a major risk factor for cardiovascular diseases (CVD), such as stroke, myocardial infarction, heart failure, and kidney disease [1]. Most morbidity and mortality related to HT occurs in older adults [2]. In Sweden, the overall prevalence of HT is 43% in men and 29% in women [3]. In the Västra Götaland region, the prevalence of diagnosed HT among individuals over 80 years of age was nearly 50% in 2017 [4]. Streit et al. described individuals over 80 years as the oldest-old [5], this definition was also applied in the current paper.

There are differences between younger and older patients with HT. Historically, HT treatment guidelines did not offer the physician much guidance on the best methods of treating elderly patients with multimorbidity, presumably because – as a recent review recognised [6] – these patients are often excluded from drug trials. The HT treatment guidelines issued by the European Society of Hypertension (ESH) [1] were updated in 2023. The revised version recommends a diagnostic work-up that accounts for both personal and medical history of the patient when making decisions about treatment for the oldest-old. However, these recommendations are not yet widely accepted, as it remains difficult for general practitioners (GPs) to assess patients’ fragility, quality of life, risk of polypharmacy and life expectancy, and to subsequently include these factors in the decision process [6]. According to the new ESH guidelines, very old patients with HT who are fit for their age, should combine antihypertensive drug treatment with lifestyle modifications, including mild physical activity [1]. Prior studies have indicated that this approach has clear benefits in terms of reducing the risk of cardiovascular complications and mortality in older patients [7,8].

The challenge for the physician is to weigh the risks of providing insufficient treatment, which may lead to cardiovascular complications [6,9], against the risks of overtreatment and polypharmacy, which may lead to adverse effects [10,11] such as acute kidney injury and syncope [12,13].

Most Swedish patients with HT, including the oldest-old, are managed in primary health care (PHC) [14]. Choosing individual treatment goals and medications is often complicated [10,15] and different choices are made by different physicians. A case-vignette survey investigated the use of guidelines among GPs from 29 countries when managing HT in the oldest-old. Female GPs and less experienced GPs used guidelines more often, although the treatment decisions ultimately made were the same, regardless of whether the GP referred to guidelines [16].

In an earlier qualitative study, we explored the considerations and experiences of Swedish GPs and GP trainees in PHC in Region Västra Götaland as they treated HT among the oldest-old [17]. In that study, this task was considered complicated due to the necessity of balancing medical and humanistic considerations. Multimorbidity, polypharmacy and the conditions of the patients’ daily lives were identified as important factors for making treatment decisions. Multimorbidity is often accompanied by an increasing impact on the patients’ living conditions. Living conditions can be described as the factors affecting the circumstances in which people live, with regard to their well-being as economy, housing, work, social contacts, and health [18]. In previous studies of elderly patients, living conditions were often described as the patient’s housing and sometimes including necessary support [19,20]. In our previous qualitative study, conditions of daily life were discussed mostly as the patient’s housing and sometimes included also how a patient desires to live the life [17].

Clinical experience and support from the PHC – for instance, access to registered nurses performing HT controls and to home blood pressure monitoring devices – were also important for the treatment decisions. The decisions were based on a balance of risks against benefits and the communication with the patients.

Against this background, the main objective of the current study was to conduct a nationwide investigation of Swedish GPs’ and GP trainees’ routines and influencing factors in the management of HT among the oldest-old. In addition, we investigated whether the respondents’ professional experience and gender affected the chosen management.

## Methods

### Study population

The sample comprised GPs and GP trainees who were (i) members of the Swedish Association of General Practice (Svensk Förening för AllmänMedicin, SFAM) and/or (ii) members of the GP Union (Distriktsläkarföreningen, DLF) and/or (iii) members of closed social media groups for Swedish GPs and GP trainees. Approximately 80% of the Swedish GPs (2504 of 3130 in 2021) and GP trainees (2632 of 3290 in 2021) [21] are members of SFAM, the GP union and/or social media groups on Facebook^®^. We estimated that, using these methods, we could reach about 5000 physicians with information about the study. The Swedish Ethical Review Authority (Reg. no. 2022-02997-01; 2022-05289-02) assessed and approved the study protocol.

### Data collection

After securing permission from the GPs responsible for managing SFAM, DLF and the social media groups, the study announcement was published in the SFAM and DLF newsletters and posted in the closed social media groups. The announcement included a link to a digital questionnaire.

Data were collected using a web-based survey (esMaker^®^NX3-V 3.0) between 8 February and 31 May 2023. The data were transferred to Microsoft^®^ Excel^®^ (Microsoft 365 MSO, version 2308). No personal identification details were requested, and no email lists were used. Only the date of the response was registered. Reminders were posted three times (after 14 days, two months, and three months) in the closed social media groups. No reminders were posted in the newsletters.

### The questionnaire

The questionnaire included 29 items, divided into four main topics: (1) physician demography and experience in PHC, (2) oldest-old patients and HT, (3) HT treatment and physician attitudes towards the various treatment options, and (4) issues with the organisation of care.

The demographic information collected on the physicians included age, gender, years as a physician in PHC [22] and location of the PHCC at which the physician worked [16]. The form also asked for the name of the Swedish region in which the PHCC was located (out of 21 optional regions).

Questions about oldest-old patients and HT included the frequency of seeing the oldest-old in the clinic, use of guidelines, and acceptable values for minimum and maximum systolic blood pressure (SBP) and diastolic blood pressure (DBP). They were formulated based on the results of our prior study [17] completed with questions similar to those in other studies in the field [22–24].

Questions about HT treatment (threshold values for treatment and choice of medication) and the physician’s attitude towards other factors influencing treatment (lifestyle factors, medical factors, risk for complications, patient’s living conditions) were also asked. One question solicited information on how the respondents prioritised medical considerations against humanistic concerns.

Finally, questions were asked about the organisation of care, such as teamwork with nurses and community care, access to different types of blood pressure monitoring, continuing education, and interprofessional discussions. Questions were also asked about suggestions for improvements of HT care and guidelines. A list of all variables, including categorisations and dichotomisations, is provided in Supplementary Appendix 1.

The questionnaire was first discussed and tested within the research team. It was then tested in several stages on physicians of the same age and gender as the target group, who had varying levels of experience with PHC, and on a GP with a native tongue other than Swedish. These test persons were excluded from the main study. The time needed to fill out the questionnaire was recorded. The tests were audio-recorded and then assessed by the research team. Minor adjustments to the wording of the questionnaire were made after the test.

### Analysis

The data were analysed using the Statistical Package for the Social Sciences (SPSS) (version 28.0.1.1, IBM^®^). Descriptive statistics calculated numbers, percentages, means, standard deviations (SDs) and ranges (min–max) when applicable. Two-sided chi-square tests were used to analyse differences in gender and work experience versus categorical variables. Independent samples t-tests were used to examine differences in the mean blood pressure values assessed as acceptable according to different characteristics of the respondents. The association between the respondents’ characteristics (explanatory variables) and their prioritisation of living conditions and medical factors when considering HT treatment for the oldest-old (the dependent variable) were analysed using bivariate logistic regression analysis. The impact of possible confounders was examined using multivariate models.

## Results

Of the estimated 5000 possible respondents, 577 (11%) started answering the digital questionnaire, and 397 questionnaires were completed, resulting in a completion rate of 69%.

Table 1 shows the characteristics of the respondents overall and by gender. Three out of five respondents were women, who were significantly younger than the male respondents (*p* < 0.001). This corresponds with the finding that long experience (≥10 years) of work in PHC is more common among men (*p* < 0.001). Most (>90%) respondents reported seeing oldest-old patients in their office frequently, and more than 50% cared for the oldest-old in nursing homes or community care. The respondents represented all 21 regions of Sweden, and half of the respondents worked in the three most populated regions: Stockholm, Västra Götaland, and Skåne (not in table).

**Table 1.** Characteristics by gender of the respondents from Swedish primary health care who completed the survey.

	Total *n* (%)	Females *n* (%)	Males *n* (%)	*p*
Physician’s age group, years				
25–34	32 (8.1)	25 (10.2)	7 (4.7)	
35–64	312 (78.8)	205 (83.3)	106 (71.2)	
65–74	36 (9.1)	13 (5.3)	23 (15.4)	
≥ 75	16 (4.0)	3 (1.2)	13 (8.7)	<0.001
Country of medical education				
Sweden	319 (80.7)	199 (80.9)	120 (80.5)	
Europe (besides Sweden)	67 (17.0)	43 (17.5)	24 (16.1)	
Outside Europe	9 (2.3)	4 (1.6)	5 (3.4)	0.665
Profession				
GP	327 (86.5)	201 (84.1)	126 (90.6)	
GP trainee	51 (13.5)	38 (15.9)	13 (9.4)	0.073
Working in Swedish primary health care, years				
<10	128 (32.4)	87 (35.4)	41 (27.5)	
10–20	159 (40.3)	113 (45.9)	46 (30.9)	
> 20	108 (27.3)	46 (18.7)	62 (41.6)	<0.001
Location of PHCC				
Rural (<5 000 inhabitants)	39 (9.9)	24 (9.8)	15 (10.1)	
Small town (≥ 5 000–< 50 000)	173 (43.9)	106 (43.1)	67 (45.3)	
Larger city (≥ 50 000–< 300 000)	99 (25.1)	66 (26.8)	33 (22.3)	
Metropolitan city (≥ 300 000)	83 (21.1)	50 (20.3)	33 (22.3)	0.920
Taking care of patients ≥ 80 years				
Seldom	24 (6.1)	13 (5.3)	11 (7.4)	
Often	371 (93.9)	233 (94.7)	138 (92.6)	0.398
Having special task for elderly ≥ 80 years				
In community care	92 (23.4)	62 (25.2)	30 (20.3)	
In nursing homes	103 (26.1)	59 (24.0)	44 (29.7)	
In intermediate care	7 (1.8)	2 (0.8)	5 (3.4)	
Not at all	192 (48.7)	123 (50.0)	69 (46.6)	0.955

GP: General Practitioner; PHCC: Primary Health Care Centre.

Missing cases: < 1.0%, except profession = 4.8%.

The mean acceptable value of the minimum SBP as assessed by the respondents was 114.5 mmHg (SD 12.6), and the mean value of the maximum accepted SBP was 152.1 mmHg (SD 9.6). As can be seen in Table 2, the physician’s age and years working in PHC were significant factors, as older and more experienced respondents accepted a higher minimum SBP than their counterparts (*p* = 0.022 and *p* = 0.010, respectively). As for maximum SBP, the PHCC being located in a rural area and respondents having a special task in elderly care, was associated with an acceptance of a higher score (*p* = 0.041 and *p* = 0.002, respectively).

**Table 2.** Acceptable values for systolic blood pressure (SBP) in the oldest-old as assessed by the respondents.

	Minimum acceptable value of systolic BP in mmHg mean (SD) min‒max	*p*	Maximum acceptable value of systolic BP in mmHg mean (SD) min‒max	*p*
All respondents	114.5 (12.6) 70‒150		152.1 (9.6) 110‒180	
Physician’s gender				
Female	114.3 (11.8) 70‒150		152.2 (9.1) 135‒180	
Male	115.0 (14.2) 80‒150	0.640	152.0 (10.5) 110‒180	0.891
Physician’s age group, years				
25–64	113.8 (12.0) 70‒150		151.9 (9.5) 110‒180	
65+	119.6 (15.7) 85‒150	0.022	153.5 (10.2) 135‒180	0.311
Country of medical education				
Sweden	114.5 (12.3) 70‒150		152.5 (9.6) 110‒180	
Outside Sweden	114.8 (14.1) 90‒150	0.884	150.6 (9.5) 130‒180	0.138
Profession				
GP	114.5 (12.4) 70‒140		151.6 (9.8) 110‒180	
GP trainee	112.4 (12.4) 90‒150	0.296	153.1 (7.5) 139‒170	0.233
Working in Swedish primary health care, years				
< 10	112.0 (13.0) 70‒150		152.4 (8.9) 110‒180	
≥ 10	115.8 (12.3) 80‒150	0.010	152.0 (10.0) 130‒180	0.692
Location of PHCC				
Rural or small town	114.1 (12.5) 80‒140		153.1 (10.2) 110‒180	
Large city or metropolitan city	115.0 (12.8) 70‒150	0.494	151.1 (8.9) 130‒170	0.041
Taking care of patients ≥ 80 years				
Seldom	112.9 (11.2) 90‒130		151.8 (7.8) 140‒165	
Often	114.6 (12.8) 70‒150	0.521	152.2 (9.8) 110‒180	0.849
Having special task for elderly ≥ 80 years				
Community care/nursing home	115.5 (13.2) 70‒150		153.7 (9.1) 130‒180	
No special task	113.5 (12.1) 80‒150	0.144	150.6 (9.9) 110‒180	0.002

SD: Standard deviation; PHCC: Primary Health Care Centre.

Missing values: 6.8–18.7%.

The mean acceptable value for minimum DBP as assessed by the respondents was 61.4 mmHg (SD 11.7), and the mean value of the maximum accepted DBP was 92.6 mmHg (SD 6.2). Differences in assessment values based on profession were observed: GP trainees and those with less experience in PHC accepted a higher maximum DBP (*p* = 0.011 and *p* = 0.013, respectively). Older physicians and GP trainees accepted a higher minimum DBP (*p* = 0.009 and *p* = 0.019, respectively) (Supplementary Table S1). Out of a total of 1349 answers, we excluded 14 outliers for accepted minimum/maximum SBP and DBP values from the analyses, as it was obvious that the respondents had confused the minimum and maximum values for SBP and DBP.

The respondents’ routine treatment of HT in the oldest-old was dominated by the prescription of antihypertensive drugs, which almost all respondents (99%) prescribed often or very often (Table 3). Lifestyle changes were recommended less often (60%). Almost all respondents stated that patient’s previous cardiovascular disease, comorbidity with diabetes, kidney function, risk of falling, patient living independently and patient’s opinion on HT treatment strongly influenced the choices of treatment (Supplementary Table S2).

**Table 3.** Respondents’ routine treatment of hypertension in oldest-old patients by gender.

	Female *n* (%)	Male *n* (%)	*p*
Most commonly used blood pressure limit to *start/intensify* antihypertensive treatment in patients > 80 years			
SBP > 160 mm Hg	240 (98.4)	142 (95.3)	0.075
SBP > 140 mm Hg	80 (32.9)	48 (32.7)	0.956
DBP > 100 mm Hg	208 (86.0)	113 (76.9)	0.022
Reasons for *discontinuing* antihypertensive treatment in patients > 80 years (very/rather common)			
SBP < 140 mm Hg	56 (23.3)	55 (37.2)	0.003
SBP < 120 mm Hg	194 (80.5)	124 (83.2)	0.501
Dizziness upon standing up	209 (86.7)	128 (87.7)	0.788
Increased falling tendency	226 (93.8)	138 (93.2)	0.835
Cognitive deterioration	123 (51.1)	86 (58.1)	0.203
Physical deterioration	124 (51.9)	82 (55.0)	0.546
Relatives wish discontinuation	65 (27.1)	58 (38.9)	0.015

SBP: Systolic blood pressure; DBP: Diastolic blood pressure.

Missing values 0–5.3%.

Most respondents (about 95%) stated that they often started or intensified drug treatment when the patient’s SBP > 160 mmHg, but only about one-third did this if the SBP > 140 mmHg, regardless of the physician’s experience or gender. Approximately four out of five respondents initiated or intensified drug therapy at DBP > 100 mmHg, and female physicians reported doing this more often than male physicians (*p* = 0.022). The most common reasons given for the discontinuation of antihypertensive treatment were dizziness, tendency to fall, and a clinically registered SBP < 120 mmHg (Table 3).

**Figure 1.**
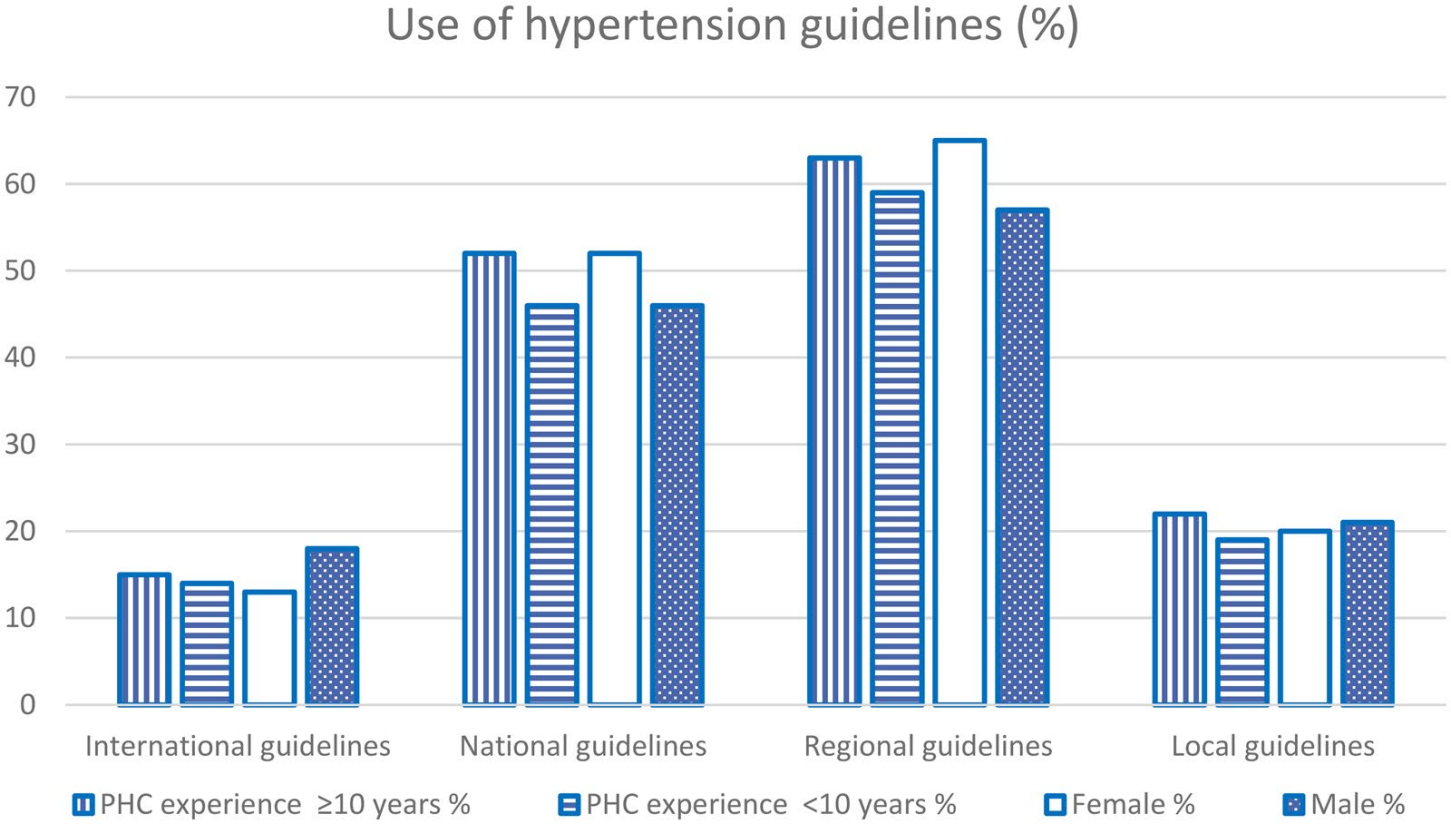
Use of hypertension treatment guidelines by experience in Swedish primary health care and by gender.

Figure 1 visualises the reported use of existing guidelines for the oldest-old, which are general for the treatment of HT in all ages. The most used guidelines were the regional guidelines, followed by the national ones. There were no statistically significant differences in guideline use by PHC experience or gender (Supplementary Table S2).

In Table 4, some attitudes of the respondents are presented. Overall, most respondents had a positive attitude towards guidelines, with about 70% identifying them as useful. They were assessed as more useful by experienced respondents than by less experienced respondents (*p* = 0.012). Both medical factors and the patient’s living conditions were identified as important influences on the choice of drug treatment by almost all respondents, but a higher proportion of female than male physicians identified living conditions as important (99.2% versus 95.9%, *p* = 0.027).

**Table 4.** Attitudes of the respondents towards guidelines and hypertension treatment in the oldest-old patients by experience in Swedish primary health care and by gender.

	PHC experience ≥ 10 years *n* (%)	PHC experience < 10 years *n* (%)	*p*	Female *n* (%)	Male *n* (%)	*p*
Assessed usefulness of guidelines when treating hypertension in patients > 80 years						
Useful	205 (77.1)	82 (65.1)		181 (74.5)	105 (70.9)	
Not useful	61 (22.9)	44 (34.9)	0.012	62 (25.5)	43 (29.1)	0.444
Overall assessment of influencing factors for choice of treatment in patients > 80 years (very/fairly important)						
Medical factors	264 (98.9)	125 (97.7)	0.354	243 (98.8)	145 (98.0)	0.527
Living conditions	261 (97.8)	126 (98.4)	0.652	244 (99.2)	142 (95.9)	0.027
If forced to choose: which are more important?						
Medical factors	70 (26.4)	22 (17.2)		49 (20.2)	43 (28.9)	
Living conditions	195 (73.6)	106 (82.8)	0.043	194 (79.8)	106 (71.1)	0.049

PHC: Primary health care.

Missing values 0.3–1.3%.

A follow-up question asked the respondents to choose which of the two factors they considered most important in making decisions about treatment. Living conditions were chosen as most important by a great majority (76.8%). Physicians with less PHC experience (*p* = 0.043) and female respondents (*p* = 0.049) chose living conditions as the most important factor more frequently than their counterparts.

The impact of possible confounders of the prioritisation of living conditions over medical factors in decision-making about HT treatment is presented in Table 5. The independent variables are based on the earlier analyses. In the multivariate analyses, adjustments were made for gender, location of PHCC and special task of caring for the oldest-old. Mostly stable associations were found – for instance, that younger physicians more often prioritised living conditions (crude odds ratio (OR): 2.84, 95% CI 1.54–5.22 and fully adjusted OR: 2.56, 95% CI 1.35–4.91). However, the association between length of experience in PHC and prioritisation of living conditions was attenuated by the adjustments (OR: 1.68, 95% CI 0.98–2.88).

**Table 5.** Probability of prioritising patient’s living conditions over patient’s medical factors depending on respondents’ gender, working experience, age and attitude towards use of guidelines in Swedish primary health care.

Explanatory variable	Grouping/rating	Total group *n* (%)	Prioritise living conditions *n* (%)	OR	95% CI
Experience in PHC	≥ 10 years = 0	268 (67.7)	195 (73.6)	1	
	< 10 years = 1	128 (32.3)	106 (82.8)	1.73^a^	1.01–2.95
				1.68^b^	0.98–2.88
Age group	≥ 65 years = 0	52 (13.1)	30 (57.7)	1	
	25–64 years = 1	344 (86.9)	271 (79.5)	2.84^a^	1.54–5.22
				2.56^b^	1.35–4.91
Usefulness of guidelines	Very/certain = 0	287 (73.2)	208 (73.2)	1	
	Little/no = 1	105 (26.8)	89 (84.8)	2.03^a^	1.12–3.68
				2.07^b^	1.13–3.78

Outcome: medical factors = 0, living conditions = 1.

^a^Crude association.

^b^Association adjusted for gender, location of PHCC and special task in care for the oldest-old.

Finally, the respondents were asked to consider organisational factors that could affect their choice of HT treatment (Table 6). Over 75% of the respondents were very interested in clearer HT treatment guidelines for the oldest-old, common medication lists being made accessible to all caregivers and increased cooperation with community health care. Cooperation and communication with other staff and with patients yielded the highest scores overall (Supplementary Table S3). Gender differences in the responses indicated that female physicians, to a greater extent than male physicians, emphasise the importance of improving the organization of HT care for the oldest-old.

**Table 6.** Suggested organisational changes for the future by gender.

	Females *n* (%)	Males *n* (%)	*p*
Clearer guidelines for treating multimorbid patients > 80 years with hypertension	217 (88.9)	100 (68.0)	<0.001
Improved cooperation with nurses and nursing staff	180 (74.1)	94 (65.7)	0.082
Common medication list for primary, hospital and community care	242 (98.8)	134 (91.2)	<0.001
Special geriatric outpatient clinics	130 (53.1)	63 (42.6)	0.044
Increased cooperation with community health care	222 (91.0)	116 (79.5)	0.001
Increased cooperation with hospital staff	138 (57.0)	67 (45.9)	0.034

Factors of importance and suggested organisational changes: very/rather important vs not very important/not important at all.

Missing values 0–2.5%.

## Discussion

### Main findings

The main findings in this study were that the respondents took both patient-specific medical factors and living conditions into account when making choices about HT treatment for the oldest-old. When the respondents were asked to choose, all agreed that living conditions were more important for all the respondents. Compared to males and more experienced respondents, female and less experienced respondents identified living conditions as significantly more important. Compared to those with less experience, respondents with longer PHC experience assessed the HT treatment guidelines for the oldest-old as more useful. The maximum acceptable SBP among the oldest-old was higher for respondents working at rural PHCCs and those who had special tasks in community care/nursing homes. Organisational factors and the improvement of HT care for the oldest-old were of significantly greater importance to female respondents.

### Discussion of the results and comparison with existing literature

SBP is a stronger predictor than DBP of coronary heart disease [25] and cardiovascular disease [26] in the oldest-old, which motivated us to focus on SBP when describing acceptable blood pressure. The respondents in our study stated a maximum SBP of 152.1 mmHg, which was slightly higher than the guidelines for HT treatment recommend [27]. This might be explained by the fact that our question concerned the whole unspecified and heterogenous group of the oldest-old from healthy individuals to patients with multimorbidity or prior side-effects and interactions between antihypertensive drugs and other medication. Respondents who had a special task caring for the elderly in nursing homes/community care accepted higher SBP values, which may be explained by their being used to meeting individuals with high levels of frailty or multimorbidity [10]. Respondents working at rural PHCCs, or in small towns accepted higher SBPs, which might be attributable to personal knowledge of the individual patients, the patients’ living conditions and to the opinions of relatives, influencing decisions about treatment.

Only just under one-third of the respondents indicated that they would initiate or intensify HT treatment in the oldest-old when the patient’s SBP exceeded the recommended level of < 140 mm Hg, contrary to what has been recommended based on results from the international Hypertension in the Very Elderly Trial (HYVET). That study showed that the oldest-old benefit from antihypertensive treatment, exhibiting reduced risks of cardiovascular events, all-cause mortality and heart failure [7]. Likewise, The Systolic Blood Pressure Intervention Trial (SPRINT) reported benefits from intensive BP control when SBP < 120 mm Hg for HT patients > 75 years, reducing cardiovascular events and death from any cause, regardless of the patient’s frailty status [28]. Our results suggest that the positive results from these previous studies are not widely known among Swedish GPs, or that they consider the risk of side effects to be more important than meeting a recommended BP target.

Although research findings and guidelines recommend lifestyle improvements [1], fewer than two-thirds of the respondents stated that they raised this issue very or fairly often with patients as a treatment choice. When specifically asked for certain lifestyle changes to suggest; exercise, smoking cessation, and reduced alcohol intake were often advised. This corresponds with previous studies and HT treatment guidelines, which demonstrate that lifestyle changes can generate a significant reduction in BP [29]. The explanation for dietary advice seldom being given may be that the respondents do not believe that oldest-old patients with long-term lifestyle habits would change their diets [30]. In order to investigate whether and how reduced ability and weakened health in the oldest-old influenced the choice of HT treatment, the respondents in the study were asked questions about i.a., patients’ comorbidities, fall susceptibility and living arrangements. Our respondents stated that these factors were of great importance, similar to GPs in an Australian study [10]. Frailty should be addressed as a topic for future research in the field [31].

In our study, the HT treatment guidelines most commonly referred to were regional and national guidelines. In a recent Swedish study [32] the guidelines reported to be most commonly used were also regional and – contrasting with our findings – local. This difference could possibly be explained by the fact that our respondents represented more Swedish regions (21 compared to 8) and that some regions are small and therefore do not write their own HT guidelines.

Previous studies have shown that less experienced physicians tended to be more adherent to guidelines [16] than more experienced physicians did [33]. Surprisingly, in our study, respondents with longer PHC experience assessed the HT guidelines for treating the oldest-old as more useful than did those with less PHC experience. Perhaps our respondents with more clinical experience had, over time, recognised the benefits of better BP control and fewer complications for the patients.

The patient’s living conditions were not explicitly defined as these could be a broad array of factors, as described in the introduction thus, we would not guide the respondents, but they could answer based on what they considered to be ‘‘living conditions’’. It is possible that it would have been better to provide a definition, but it was difficult precisely because it is such a broad concept, and various interpretations have been made in different contexts. Our findings that the respondents considered living conditions more important than medical factors for making HT treatment choices might reveal that the respondents rated present quality of life higher than the potential risk of future medical complications from undertreated HT. That is, they might have considered that possible side effects of HT medication, such as risk for syncope, acute kidney injury and risk of falling, can influence a patient’s everyday quality of life negatively [12,13].

Some of the results in our study showed gender differences, especially in attitudes towards and views on organisational issues. These differences can probably be explained by both sociocultural and professional causes [34]. Thus, expectations for female physicians to be more responsive and caring may be higher than those for male physicians [35]. Likewise, this may explain the female respondents’ greater tendency to consider the patient’s living conditions as more important. The choice of specialty may also be socioculturally influenced, and the higher proportion of female respondents in our study reflects the official statistics for GPs in PHC [21]. However, we found no gender differences in the use of guidelines, in contrast to an international study, wherein female GPs more often reported using guidelines [16]. The female respondents’ more frequent advocacy for changes in HT care, continuous education, and organisational changes is consistent with a study conducted in Israel [36]. The propensity for change may decrease with age, which could explain why the female physicians, who were, on average, younger than the male respondents, more often indicated a need and desire for organisational changes. The respondents would likely experience improvements if they were allowed to set aside time for professional discussions about HT treatment of the oldest-old.

### Strengths and limitations

A strength of the study was that the development of the questionnaire was based on results from our previous qualitative study [17] in combination with questions from existing literature in the field [16,22–24] and that the questionnaire was tested in several steps.

A limitation was that no mailing lists were permitted to be used due to the General Data Protection Regulation (GDPR) [37]. Therefore, it was not feasible to reach out individually to possible respondents. We used the available routes of announcing the study in professional newsletters and closed social media groups for GPs and GP trainees, which diminished the risk of coverage error [38]. Those who were not members of SFAM, DLF and/or social media groups were not reachable, which was a drawback. Response rate can be measured in different ways, and based on the estimated number that could be reached *via* the contact channels used it was very low. However, among those who actually started responding to the digital survey, the so-called completion rate was acceptable [38]. Also, there was a good representation of respondents from all 21 regions in Sweden, and the distribution by gender and age matched the official statistics [21].

Some selection bias may have occurred, as physicians with a greater interest in the subject may have been more likely to participate in the study. It is possible that non-participants would have answered the questions differently, but both ethical considerations and circumstances as well as practical reasons prevented such an analysis. It is conceivable that a less positive attitude towards the topic would have emerged among non-participants however, the extent of this selection bias is difficult to estimate. The representativeness of the study for the target group of primary care physicians must therefore be interpreted with caution.

Another possible bias was that the physicians’ stated values – for example, regarding the thresholds for SBP and DBP – might not have corresponded with what they actually do clinically [16]. The respondents did not have the possibility to tick an answer option ‘it depends’ when stating maximum and minimum SBP and DBP, but were given the opportunity to make a free-text comment.

Generally, the internal dropout rate was low. However, the low response rate is notable for the question concerning profession, possibly due to a perceived risk of disclosure of identities, as some regions had only a few GPs in each age group [21].

## Conclusion

Both medical factors and living conditions were important for GPs and GP trainees making decisions about HT treatment decisions for the oldest-old. Compared to male and more experienced respondents, female and less experienced respondents in PHC considered patients’ living conditions to be more important than medical factors. Compared to male respondents, females attributed greater importance to clinical support, cooperation with other health care providers and future organisational changes in HT care for the oldest-old.

## Supplementary Material

Supplementary Table S1 Assessed acceptable values for blood pressure_resubmit.docx

Appendix 1_resubmit.docx

Supplementary Table S3 Organisational factors revised.docx

Supplementary Table S2 Routines revised.docx

## Data Availability

The data that support the findings of this study are not openly available for reasons of sensitivity but are available from the corresponding author upon reasonable request.
