# Supplementary material for: Hypertension management in the oldest-old: a survey of physicians in Swedish primary health care
Source: Scand J Prim Health Care. 2025 Aug 25;44(1):1–12. doi: 10.1080/02813432.2025.2549088 (PMC12918363; doi:10.1080/02813432.2025.2549088)
Supplement: Supplementary Table S1 Assessed acceptable values for blood pressure_resubmit.docx [file IPRI_A_2549088_SM4023.docx]

**Supplementary Table S1. Acceptable values for blood pressure (BP) in mmHg as assessed by the survey respondents.**

|  | **Maximum** acceptable value of  **systolic** BP  mean (SD)  min‒max | p | **Maximum** acceptable value of  **diastolic** BP  mean (SD)  min‒max | p |  | **Minimum** acceptable value of  **systolic** BP  mean (SD)  min‒max | p | **Minimum** acceptable value of  **diastolic** BP  mean (SD)  min‒max | p |
| --- | --- | --- | --- | --- | --- | --- | --- | --- | --- |
| All respondents | 152.1 (9.6)  110‒180 |  | 92.6 (6.2)  75‒120 |  |  | 114.5 (12.6)  70‒150 |  | 61.4 (11.7)  0‒95 |  |
| Physician’s gender |  |  |  |  |  |  |  |  |  |
| Female | 152.2 (9.1)  135‒180 |  | 92.4 (5.9)  79‒110 |  |  | 114.3 (11.8)  70‒150 |  | 61.1 (9.8)  0‒85 |  |
| Male | 152.0 (10.5)  110‒180 | 0.891 | 93.0 (6.7)  75‒120 | 0.361 |  | 115.0 (14.2)  80‒150 | 0.640 | 62.0 (14.6)  0‒95 | 0.572 |
| Physician’s age group, years |  |  |  |  |  |  |  |  |  |
| 25-64 | 151.9 (9.5)  110‒180 |  | 92.7 (6.3)  75‒120 |  |  | 113.8 (12.0)  70‒150 |  | 60.5 (10.9)  0‒90 |  |
| 65+ | 153.5 (10.2)  135‒180 | 0.311 | 91.9 (5.2)  80‒105 | 0.371 |  | 119.6 (15.7)  85‒150 | 0.022 | 67.8 (15.3)  6‒95 | 0.009 |
| Country of medical education |  |  |  |  |  |  |  |  |  |
| Sweden | 152.5 (9.6)  110‒180 |  | 92.6 (6.2)  75‒120 |  |  | 114.5 (12.3)  70‒150 |  | 62.2 (12.1)  0‒95 |  |
| Outside Sweden | 150.6 (9.5)  130‒180 | 0.138 | 92.8 (6.0)  80‒110 | 0.769 |  | 114.8 (14.1)  90‒150 | 0.884 | 62.5 (10.0)  40‒85 | 0.404 |
| Profession |  |  |  |  |  |  |  |  |  |
| GP | 151.6 (9.8)  110‒180 |  | 92.3 (6.3)  75‒120 |  |  | 114.5 (12.4)  70‒140 |  | 60.8 (12.3)  0‒95 |  |
| GP trainee | 153.1 (7.5)  139‒170 | 0.233 | 94.5 (5.4)  79‒100 | 0.011 |  | 112.4 (12.4)  90‒150 | 0.296 | 63.8 (6.3)  50‒80 | 0.019 |
| Working in Swedish primary health care, years |  |  |  |  |  |  |  |  |  |
| < 10 | 152.4 (8.9)  110‒180 |  | 93.8 (6.0)  79‒110 |  |  | 112.0 (13.0)  70‒150 |  | 60.9 (9.0)  40‒80 |  |
| ≥ 10 | 152.0 (10.0)  130‒180 | 0.692 | 92.0 (6.2)  75‒120 | 0.013 |  | 115.8 (12.3)  80‒150 | 0.010 | 61.7 (13.0)  0‒95 | 0.564 |
|  |  |  |  |  |  |  |  |  |  |
|  |  |  |  |  |  |  |  |  |  |
|  |  |  |  |  |  |  |  |  |  |
| Location of PHCC |  |  |  |  |  |  |  |  |  |
| Rural or small town | 153.1 (10.2)  110‒180 |  | 93.2 (6.5)  75‒120 |  |  | 114.1 (12.5)  80‒140 |  | 61.1 (12.2)  0‒90 |  |
| Large city or Metropolitan city | 151.1 (8.9)  130‒170 | 0.041 | 92.0 (5.8)  79‒110 | 0.136 |  | 115.0 (12.8)  70‒150 | 0.494 | 61.8 (11.2)  0‒95 | 0.566 |
| Taking care for patients ≥ 80 years |  |  |  |  |  |  |  |  |  |
| Seldom | 151.8 (7.8)  140‒165 |  | 94.3 (3.7)  90‒100 |  |  | 112.9 (11.2)  90‒130 |  | 62.7 (6.0)  50‒70 |  |
| Often | 152.2 (9.8)  110‒180 | 0.849 | 92.5 (6.3)  75‒120 | 0.065 |  | 114.6 (12.8)  70‒150 | 0.521 | 61.4 (11.9)  0‒95 | 0.471 |
| Having special task for  elderly ≥ 80 years |  |  |  |  |  |  |  |  |  |
| Community care/nursing home | 153.7 (9.1)  130‒180 |  | 93.0 (6.1)  79‒110 |  |  | 115.5 (13.2)  70‒150 |  | 61.5 (11.0)  6‒90 |  |
| No special task | 150.6 (9.9)  110‒180 | 0.002 | 92.2 (6.3)  75‒120 | 0.218 |  | 113.5 (12.1)  80‒150 | 0.144 | 61.4 (12.4)  0‒95 | 0.966 |

SD = Standard deviation

PHCC = Primary Health Care Centre

Missing values: 6.1-15.4%, except min DBP: 25.6-30.3%
