# Supplementary material for: Hypertension management in the oldest-old: a survey of physicians in Swedish primary health care
Source: Scand J Prim Health Care. 2025 Aug 25;44(1):1–12. doi: 10.1080/02813432.2025.2549088 (PMC12918363; doi:10.1080/02813432.2025.2549088)
Supplement: Appendix 1_resubmit.docx [file IPRI_A_2549088_SM4022.docx]

**Appendix 1. Questionnaire items.**

| **Items** | **Answer options and dichotomisations when applicable** |
| --- | --- |
| *Demography* |  |
| Gender | woman, man, other |
| Age group (years) | 25‒34/35‒64 *versus* 65‒74/≥75 |
|  |  |
| *Professional characteristics* |  |
| Country of medical education | Sweden *versus* Europe/outside Europe |
| Currently working in primary health care (PHC) | yes *versus* no |
| Professional level | GP trainee, GP, other |
| Experience as a physician (years) | <10 *versus* 10‒20/≥20 |
| Experience in PHC (years) | <10 *versus* 10‒20/≥20 |
| Working in Swedish region (name) | 21 regions of Sweden |
| Location of primary health care centre (PHCC) | rural area/small town *versus* larger city/metropolitan city |
|  |  |
| *Oldest-old patients and hypertension* |  |
| Frequency of seeing oldest-old patients at the PHCC | never/seldom/sometimes *versus* fairly/very often |
| Having special task in taking care of the oldest-old | community/intermediate care/nursing home *versus* not at all |
| Estimated proportion of participant’s oldest-old patients with hypertension who have 2 or more diseases | < 25%/25‒50% *versus* > 50% |
| Guidelines used for treatment decisions about the oldest-old with hypertension:   - International guidelines - National guidelines - Regional guidelines - Local guidelines - Own clinical experience | Very often/fairly often *versus* sometimes/fairly seldom/never |
| Perceived benefit of existing guidelines for the treatment of hypertension in the oldest-old | great benefit/some benefit *versus* little benefit/no benefit |
| Treatment target for SBP in oldest-old | minimum SBP (value in mmHg)  maximum SBP (value in mmHg) |
| Treatment target for DBP in oldest-old | minimum DBP (value in mmHg)  maximum DBP (value in mmHg) |
|  |  |
| *Hypertension treatment and physician’s attitudes towards treatment options* |  |
| Use of hypertension treatment:   - lifestyle recommendations - antihypertensive drugs | very often/often *versus* seldom/never |
| Extent of use of lifestyle recommendations:   - diet - exercise - smoking - alcohol | very often/often *versus* seldom/never |
| Importance of patient’s medical history when starting antihypertensives:   - cardiovascular disease (CVD) - diabetes - kidney function - cognitive impairment/dementia - glaucoma - men: prostatism - risk factors CVD | very important/important *versus* not very important/not important at all |
| Important patient factors when starting antihypertensives:   - biological age - opinion on treatment - risk of falling - high alcohol consumption - previous side effects of antihypertensives | very important/important *versus* not very important/not important at all |
| Importance of factors when treating hypertension:   - medical factors - living conditions | very important/important *versus* not very important/not important at all |
| Most important factors if forced to choose | medical factors; living conditions |
| In an 85-year-old patient, likelihood of starting antihypertensives based on the patient’s form of residence:   - own residence - nursing home | starting/likely to start *versus* likely not to start/not starting |
| Which antihypertensives are the first, second and third choices for treatment of oldest-old:   - ACE-inhibitor - ARB - betablocker - calcium antagonist - thiazide - MRA | First choice; second choice; third choice |
| Blood pressure thresholds for starting or intensifying antihypertensives:   - SBP > 160 mmHg - SBP > 140 mmHg - DBP > 100 mm Hg | very often/often *versus* seldom/never |
| Terminating antihypertensives:   - SBP < 140 mmHg - SBP < 120 mmHg - patient has dizziness - patient is falling - cognitive deterioration - physical deterioration - relatives wish discontinuation | very often/often *versus* seldom/never |
|  |  |
| *Organisation of care* |  |
| Factors of importance in hypertension treatment for the oldest-old:   - Teamwork with nurse - cooperation with community care - access to home blood pressure device - access to ambulatory blood pressure monitoring - continuous education - reading scientific publications - discussion with other GPs - access to colleague specialised in hypertension - follow-up visit after change of medication - yearly check-ups | very important/important *versus* not very important/not important at all |
| Importance of communication about hypertension treatment with:   - patient - relatives - community caregivers - caregivers at nursing home - hospital physicians | very important/important *versus* not very important/not important at all |
| Organisational improvements to hypertension care for the oldest-old:   - guidelines for older patients with co-morbidity - increased teamwork with nurses and nursing staff at PHCC - common medication list - geriatric outpatient clinics - increased cooperation with community care/hospital | very important/important *versus* not very important/not important at all |

ACE-inhibitor: angiotensin-converting enzyme inhibitor

ARB: angiotensin 2 receptor blocker

MRA: mineralocorticoid receptor antagonist
