# Supplementary material for: Hypertension management in the oldest-old: a survey of physicians in Swedish primary health care
Source: Scand J Prim Health Care. 2025 Aug 25;44(1):1–12. doi: 10.1080/02813432.2025.2549088 (PMC12918363; doi:10.1080/02813432.2025.2549088)
Supplement: Supplementary Table S3 Organisational factors revised.docx [file IPRI_A_2549088_SM4021.docx]

**Supplementary Table S3. Organisational factors that influence treatment of hypertension in oldest-old patients and suggestions for organisational changes in the future by experience in Swedish primary health care and by gender.**

|  | PHC experience  ≥ 10 years  n (%) | PHC experience  <10 years  n (%) | *p* | Females  n (%) | Males  n (%) | *p* |
| --- | --- | --- | --- | --- | --- | --- |
| **Factors of importance for antihypertensive treatment** |  |  |  |  |  |  |
| Teamwork with nurses | 227 (84.7) | 104 (81.9) | 0.479 | 208 (84.9) | 122 (81.9) | 0.431 |
| Cooperation with nursing staff | 234 (88.0) | 103 (80.5) | 0.048 | 215 (87.8) | 121 (81.8) | 0.102 |
|  |  |  |  |  |  |  |
| Access to home blood pressure device | 208 (77.9) | 105 (82.7) | 0.274 | 196 (80.0) | 116 (78.4) | 0.701 |
| Access to ambulatory blood pressure monitoring | 172 (64.2) | 70 (54.7) | 0.070 | 161 (65.4) | 81 (54.4) | 0.029 |
|  |  |  |  |  |  |  |
| Opportunity for continuous education | 215 (80.5) | 101 (78.9) | 0.707 | 214 (87.0) | 102 (68.9) | <0.001 |
| Opportunity to read scientific publications | 138 (51.9) | 61 (47.7) | 0.433 | 128 (52.0) | 71 (48.3) | 0.474 |
| Discussion with other GPs | 245 (92.1) | 115 (89.8) | 0.455 | 233 (95.1) | 126 (85.1) | <0.001 |
| Access to GP colleague specialised in hypertension | 55 (20.7) | 39 (30.7) | 0.029 | 58 (23.7) | 36 (24.5) | 0.855 |
| Follow-up visit with patient after change in medication | 235 (87.7) | 112 (88.2) | 0.887 | 215 (87.8) | 131 (87.9) | 0.961 |
| Yearly check-ups of patients | 248 (92.5) | 112 (88.2) | 0.156 | 231 (94.3) | 128 (85.9) | 0.005 |
| Communication with the patient | 266 (99.3) | 127 (99.2) | 0.970 | 244 (99.2) | 148 (99.3) | 0.875 |
| Communication with relatives | 207 (78.1) | 98 (76.6) | 0.730 | 198 (80.8) | 106 (72.1) | 0.046 |
| Communication with nurses (i.e. community nurses) | 260 (97.4) | 121 (94.5) | 0.153 | 239 (97.6) | 141 (94.6) | 0.129 |
| Communication with assistant nurses | 218 (82.3) | 95 (74.8) | 0.085 | 199 (82.2) | 113 (75.8) | 0.127 |
| Communication with senior physicians at the hospital | 56 (20.9) | 43 (33.9) | 0.006 | 62 (25.2) | 37 (25.0) | 0.964 |
| **Suggested organisational changes for the future** |  |  |  |  |  |  |
| Clearer guidelines for treating multimorbid  patients > 80 years with hypertension | 211 (79.6) | 107 (84.3) | 0.274 | 217 (88.9) | 100 (68.0) | <0.001 |
| Improved cooperation with nurses and nursing staff | 184 (70.5) | 91 (72.2) | 0.726 | 180 (74.1) | 94 (65.7) | 0.082 |
|  |  |  |  |  |  |  |
| Common medication list for primary, hospital and  community care | 252 (95.1) | 125 (97.7) | 0.229 | 242 (98.8) | 134 (91.2) | <0.001 |
| Special geriatric outpatient clinics | 123 (46.2) | 70 (54.7) | 0.117 | 130 (53.1) | 63 (42.6) | 0.044 |
| Increased cooperation with community health care | 227 (86.3) | 112 (87.5) | 0.746 | 222 (91.0) | 116 (79.5) | 0.001 |
| Increased cooperation with hospital staff | 132 (50.6) | 74 (57.8) | 0.180 | 138 (57.0) | 67 (45.9) | 0.034 |

Factors of importance and suggested organisational changes: very/rather important vs not very important/not important at all.

Missing values: 0‒2.5%
