# Supplementary material for: Hypertension management in the oldest-old: a survey of physicians in Swedish primary health care
Source: Scand J Prim Health Care. 2025 Aug 25;44(1):1–12. doi: 10.1080/02813432.2025.2549088 (PMC12918363; doi:10.1080/02813432.2025.2549088)
Supplement: Supplementary Table S2 Routines revised.docx [file IPRI_A_2549088_SM4020.docx]

**Supplementary Table S2. Respondents’ routine treatment of hypertension in oldest-old patients by experience in Swedish primary health care and by gender.**

|  | PHCC experience  ≥10 years  n (%) | PHCC experience  <10 years  n (%) | *p* | Total  N (%) | Female  n (%) | Male  n (%) | *p* |
| --- | --- | --- | --- | --- | --- | --- | --- |
| Use of guidelines (very or fairly often) |  |  |  |  |  |  |  |
| International guidelines | 39 (15.2) | 17 (13.5) | 0.651 | 56 (14.7) | 31 (13.0) | 25 (17.5) | 0.235 |
| National guidelines | 136 (51.9) | 58 (45.7) | 0.249 | 194 (50.0) | 126 (52.3) | 68 (46.3) | 0.250 |
| Regional guidelines | 166 (63.1) | 74 (58.7) | 0.405 | 239 (61.6) | 156 (64.5) | 83 (56.8) | 0.136 |
| Local guidelines | 54 (21.5) | 23 (18.5) | 0.504 | 77 (20.6) | 47 (20.2) | 30 (21.3) | 0.798 |
| Treatment choice for hypertension in  patients > 80 years (very/fairly often) |  |  |  |  |  |  |  |
| Lifestyle habits | 162 (61.1) | 72 (56.3) | 0.356 | 234 (59.7) | 152 (61.8) | 82 (56.2) | 0.273 |
| Antihypertensives | 263 (99.6) | 126 (98.4) | 0.208 | 388 (99.2) | 243 (99.2) | 145 (99.3) | 0.886 |
| Type of lifestyle recommendation (very/fairly often) |  |  |  |  |  |  |  |
| Dietary advice | 108 (41.7) | 52 (40.6) | 0.840 | 160 (41.5) | 102 (41.6) | 58 (41.1) | 0.924 |
| Exercise advice | 211 (81.2) | 107 (83.6) | 0.557 | 318 (82.2) | 197 (80.4) | 121 (85.2) | 0.235 |
| Smoking cessation | 234 (90.0) | 117 (91.4) | 0.658 | 351 (90.7) | 223 (91.0) | 128 (90.1) | 0.774 |
| Reduced alcohol consumption | 217 (82.8) | 108 (84.4) | 0.700 | 325 (83.5) | 209 (85.0) | 116 (81.1) | 0.325 |
|  |  |  |  |  |  |  |  |
| Most commonly used blood pressure limit to *start/intensify* antihypertensive treatment in patients > 80 years |  |  |  |  |  |  |  |
| SBP* > 160 mm Hg | 258 (97.0) | 125 (97.7) | 0.708 | 382 (97.2) | 240 (98.4) | 142 (95.3) | 0.075 |
| SBP*> 140 mm Hg | 91 (34.5) | 37 (29.1) | 0.293 | 128 (32.8) | 80 (32.9) | 48 (32.7) | 0.956 |
| DBP**> 100 mm Hg | 224 (85.2) | 98 (77.2) | 0.051 | 321 (82.5) | 208 (86.0) | 113 (76.9) | 0.022 |
|  |  |  |  |  |  |  |  |
| Reasons for *discontinuing* antihypertensive treatment in patients > 80 years (very/rather common) |  |  |  |  |  |  |  |
| SBP* <140 mm Hg | 79 (30.2) | 32 (25.2) | 0.311 | 111 (28.6) | 56 (23.3) | 55 (37.2) | 0.003 |
| SBP* <120 mm Hg | 215 (81.4) | 104 (81.9) | 0.914 | 318 (81.5) | 194 (80.5) | 124 (83.2) | 0.501 |
| Dizziness upon standing up | 231 (87.8) | 106 (84.8) | 0.409 | 337 (87.1) | 209 (86.7) | 128 (87.7) | 0.788 |
| Increased falling tendency | 242 (92.0) | 123 (96.9) | 0.068 | 364 (93.6) | 226 (93.8) | 138 (93.2) | 0.835 |
| Cognitive deterioration | 144 (55.0) | 66 (52.4) | 0.633 | 209 (54.0) | 123 (51.1) | 86 (58.1) | 0.203 |
| Physical deterioration | 146 (55.7) | 61 (48.0) | 0.154 | 206 (53.1) | 124 (51.9) | 82 (55.0) | 0.546 |
| Relatives wish discontinuation | 83 (31.7) | 40 (31.3) | 0.932 | 123 (31.6) | 65 (27.1) | 58 (38.9) | 0.015 |
|  |  |  |  |  |  |  |  |
| Patient characteristics that influence choices of treatment (very/fairly important) |  |  |  |  |  |  |  |
| Previous cardiovascular disease (CVD) | 265 (99.3) | 127 (99.2) | 0.973 | 391 (99.2) | 245 (99.6) | 146 (98.6) | 0.297 |
| Comorbidity with diabetes | 265 (99.6) | 127 (99.2) | 0.596 | 391 (99.5) | 244 (99.6) | 147 (99.3) | 0.718 |
| Kidney function | 267 (100.0) | 128 (100.0) | - | 394 (100.0) | 246 (100.0) | 148 (100.0) | - |
| Comorbidity with dementia/cognitive  impairment | 246 (92.1) | 113 (88.3) | 0.214 | 358 (90.9) | 226 (91.9) | 132 (89.2) | 0.372 |
| Comorbidity with glaucoma | 117 (44.3) | 56 (44.1) | 0.967 | 173 (44.4) | 114 (46.7) | 59 (40.4) | 0.225 |
| Men: comorbidity with prostate  hyperplasia | 135 (50.8) | 49 (38.6) | 0.024 | 184 (46.9) | 126 (51.6) | 58 (39.2) | 0.017 |
| Other risk factors for CVD | 222 (83.1) | 106 (82.8) | 0.934 | 327 (83.0) | 200 (81.3) | 127 (85.8) | 0.249 |
|  |  |  |  |  |  |  |  |
| Biological age | 238 (88.8) | 116 (90.6) | 0.583 | 353 (89.4) | 222 (90.2) | 131 (87.9) | 0.468 |
| Patient’s opinion | 263 (98.5) | 124 (96.9) | 0.283 | 386 (98.0) | 241 (98.4) | 145 (97.3) | 0.473 |
| Risk of falling | 256 (95.5) | 124 (97.6) | 0.305 | 379 (96.2) | 237 (96.7) | 142 (95.3) | 0.472 |
| Risky alcohol consumption | 249 (93.3) | 113 (88.3) | 0.095 | 361 (91.6) | 232 (94.3) | 129 (87.2) | 0.013 |
| Previous adverse effects of antihypertensives | 247 (92.9) | 117 (91.4) | 0.612 | 364 (92.6) | 229 (93.5) | 135 (91.2) | 0.409 |
| Patient living independently | 257 (97.0) | 124 (97.6) | 0.713 | 380 (97.2) | 241 (98.4) | 139 (95.2) | 0.068 |
| Patient living in nursing home | 218 (82.6) | 103 (81.1) | 0.722 | 320 (82.1) | 205 (83.7) | 115 (79.3) | 0.278 |

*SBP = Systolic blood pressure
**DBP = Diastolic blood pressure

Missing values in table: 0-5.3%
